# A Lightweight Deep Network for Defect Detection of Insert Molding Based on X-ray Imaging

**DOI:** 10.3390/s21165612

**Published:** 2021-08-20

**Authors:** Benwu Wang, Feng Huang

**Affiliations:** 1 College of Metrology & Measurement Engineering, China Jiliang University, Hangzhou 310018, China; s1902080436@cjlu.edu.cn; 2 School of Mechanical & Energy Engineering, Zhejiang University of Science & Technology, Hangzhou 310023, China; 3 State Key Laboratory of Fluid Power and Mechatronic Systems, Zhejiang University, Hangzhou 310005, China

**Keywords:** insert molding, defect detection, intelligent manufacturing, deep learning

## Abstract

Aiming at the abnormality detection of industrial insert molding processes, a lightweight but effective deep network is developed based on X-ray images in this study. The captured digital radiography (DR) images are firstly fast guide filtered, and then a multi-task detection dataset is constructed using an overlap slice in order to improve the detection of tiny targets. The proposed network is extended from the one-stage target detection method of yolov5 to be applicable to DR defect detection. We adopt the embedded Ghost module to replace the standard convolution to further lighten the model for industrial implementation, and use the transformer module for spatial multi-headed attentional feature extraction to perform improvement on the network for the DR image defect detection. The performance of the proposed method is evaluated by consistent experiments with peer networks, including the classical two-stage method and the newest yolo series. Our method achieves a mAP of 93.6%, which exceeds the second best by 3%, with robustness sufficient to cope with luminance variations and blurred noise, and is more lightweight. We further conducted ablation experiments based on the proposed method to validate the 32% model size reduction owing to the Ghost module and the detection performance enhancing effect of other key modules. Finally, the usability of the proposed method is discussed, including an analysis of the common causes of the missed shots and suggestions for modification. Our proposed method contributes a good reference solution for the inspection of the insert molding process.

## 1. Introduction

Insert molding is a technology that can embed other materials such as metals into plastics during the injection molding process [1]. It has been extensively used in the automotive, medical, electronics, connector and other industries due to its flexibility in product design. As the structure of the mold is often complex, coupled with the fact that the thermal expansion coefficients of the embedded material and plastic are not consistent, it will be internal stress-prone and may cause defects such as deformation inside the product. Therefore, effective defect detection is critical to ensure the conformity of injection-molded parts, which can reduce or even avoid accidents caused by manufacturing defects or poor product quality. X-ray imaging-based digital radiography (DR) [2] provides an effective non-destructive detecting method, by virtue of its fast penetration, high spatial resolution, low noise and low radiation exposure. It can image the inside of injection-molded workpieces for defect inspection.

Currently, many manufacturers still diagnose the defects of DR images manually, which relies on the examiner’s subjective consciousness and mental state, and may easily lead to problems such as low efficiency and false drop [3]. Recently, machine vision has been popularly used in the defect detection of industrial products instead of labor. However, most studies are focused on the detection of products’ external surface [4,5,6,7], while few are reported on the internal defects of injection-molded parts with DR imaging. Early work in internal defect detection with DR images using machine vision was based on traditional image processing for automated supervision and localization of defects, which mainly relies on manually produced feature extractors, such as area feature extraction, edge detection, threshold segmentation [8]. Zhao et al. [9] used an improved WALBP for DR images of a bolster to effectively extract texture features of defects, and obtained a higher recognition rate than the basic WLBP algorithm, but the algorithm design is complex and the recall of cracks also needs to be improved. Xiao et al. [10] analyzed the texture features of DR images of aero-engine turbine blades and divided them into blocks, used the regional gray distribution function to weaken the interference of background texture on defects, and set a gray threshold for segmentation. Their segmentation results are often closely related to the selection of thresholds, and performance is not stable. These detection methods, mostly based on predefined manual features (e.g., statistical, structural, and spectral features), require high quality image acquisition and are not robust to light unevenness and noise interference. Additionally, when the design pattern is unknown, it is often necessary to adjust the model parameters or even redesign the detection scheme.

With the development of deep convolutional neural networks (CNN) and the improvement of graphics computing power, deep learning defect detection methods based on object detection are gradually being applied and going beyond traditional image processing methods [11,12,13]. There are two directions according to the stages of realization: (1) target detection framework based on region extraction. This method is based on the regional convolutional neural network (RCNN), such as fast RCNN, faster RCNN, and mask RCNN [14,15,16,17]. It can perform multiple tasks such as detection and segmentation, and has good flexibility and precision advantages, but it experiences a long inference time. In addition to some similar surface defect detections, RCNN has also launched its application on DR of injection-molded parts. Cai et al. [13], based on mask RCNN, trained the model with DR images after denoising, and obtained a better hierarchical detection effect, but the accuracy performance still needs to be improved to be actually used. (2) The regression-based target detection framework is dominated by the “you only look once” (yolo) series [18,19,20,21] and the single shot multibox detector (SSD) [22], which streamlines the feature extraction process to obtain a faster speed, but with accuracy slightly lacking in the same period of development. Combined with specific module design, this one-stage approach can often be efficiently applied to defect detection [23].

The above surveyed literatures show that the defect detection for DR images under deep learning methods is not well studied, which is still a topic worth exploring to promote its practical use. Combining the complexity of the structure of the injection-molded workpiece and the specificity of the defect itself, we summarize the difficulties of the DR defect detection task as follows. (1) Owing to the scattering caused by X-ray imaging during the penetration process, the original image carries noise and atomization characteristics, and the texture and defects in part of the background are difficult to distinguish. This difficulty is exacerbated by the complex contours. (2) Defect targets are dominated by tiny objects, such as metal wire, molten tin, plating and spots. This also belongs to the bottleneck of target detection. (3) As the complexity of the processing environment, the data collected from different camera loci generally have problems such as uneven brightness and angular deviation. (4) Some projects already in use are cumbersome and large, as reflected in model size and inference speed, which are hindrances in algorithm promotion and implementation.

Considering the above challenges, this paper proposes an algorithm for DR image defect detection based on the improved yolov5. Firstly, the statistical characteristics of DR data were analyzed, and image preprocessing with denoising and enhancement was carried out. The main pre-processing techniques included fast guided filtering of DR images to preserve features at edges, and overlapping to cut image blocks to enrich the dataset. For multi-scale targets, we added a feature detection layer of a pyramid, and embedded a transformer attention module to cope with the self-extraction and feature fusion of defect features. Moreover, to obtain a lightweight network for fast inference and easy deployment, the Ghost module was introduced instead of common convolution to reduce the network parameters while maintaining effective feature extraction. The main contributions of this work are summarized as follows:

(1) For the identification of the internal quality of the insert molding, we used a deep learning approach in computer vision, which has not often been attempted before our investigation. A complete set of inspection algorithms is established to provide processing methods for the detection of abnormalities in the insert molding.

(2) Aiming at the unsatisfactory detection of tiny targets, we use multi-task detection data set based on “image overlap”, which effectively reduces the problems of missing detection and excessive deviation of defect scale. Meanwhile, a redefinition of the positive sample bounding box is used to accelerate training convergence and mitigate the influence of multiple scales on loss divergence.

(3) In consistent environmental experiments, the advantages and limitations of various classical target detection networks for defect detection are compared and analyzed. Using yolov5 as the basis, we enhanced the feature extraction and multi-scale detection of the network, and shrank the training parameters, and obtained superior performance for the DR multi-task hybrid dataset of an injection molding workpiece.

## 2. Data and Pre-Processing

**Figure 1:**
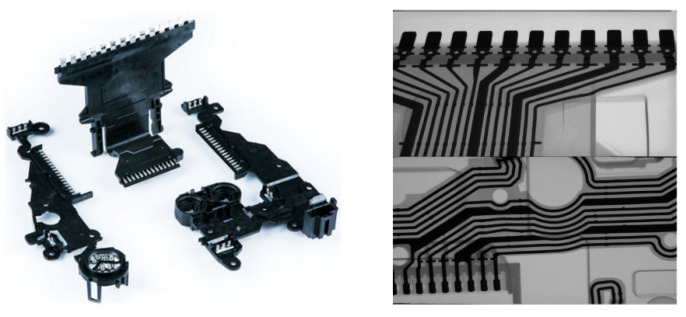
Lead frame over-molded (**left**) and its DR image under X-ray (**right**).

We collected 1522 DR images of defective insert molding workpieces. Shown on the left of Figure 1 are some sample products of molded workpieces (*CWB Company*, *Wenzhou, China*). The original image without the adjustment of window level and width contains a lot of noise due to the differences in structure, the diversity of grayscale shapes and the sizes of defect targets. Pre-processing of the images is required to ensure reliable training data, so as to distinguish texture interference from the defect itself.

### 2.1. Image Denoising

**Figure 2:**
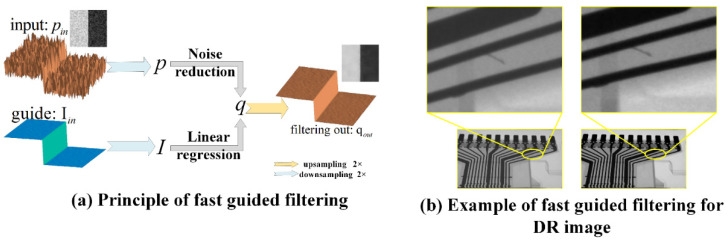
Fast guided filter principle (**a**) and its DR application example (**b**).

After normalizing the window level and width of the image, the global interference comes from slight atomization and digital noise in the collected DR data image. Some isotropic filters such as box filter and Gaussian filter can smooth the noise but erase some details at the same time, so we used the fast guided filter with edge preserving function to smooth the image [24,25]. While filtering the noise, it retains the important texture and details in the image as much as possible, and the time complexity of the algorithm is low. The principle of fast guided filtering is shown in Figure 2a.

Let the input image be *p_in_* and the filter guide image be *I_in_*, and their 2× down sampling are *p* and *I*, respectively. *q_out_* is the 2× up sampling of the filtered output *q*. In general translation transform filtering, the linear relationship between the output *q* and the input *p* is expressed as Formula (1).

(1) $$q_{i}=\sum_{j}W_{ij}(I)p_{j}$$

where the filter kernel *W* is a function of the guide map *I*. Let *w_k_* be the filter window of radius *r*, and let (*a_k_*, *b_k_*) be the constant coefficient when the center of the window lies at *k* and is uniquely determined. Assume that there is a local linear relationship between *I* and *q* in a window centered on pixel *k* and that the output will only exhibit edge characteristics if there are edges in the guided map, as expressed in Equation (2).

(2) $$q_{i}=a_{k}I_{i}+b_{k},\forall i\in w_{k}$$

Let *q_i_* be the output of *n_i_* after removing noise *n_i_* (non-edge uneven area), denoted as $q_{i}=p_{i}-n_{i}$. To minimize the discrepancy between the input and output, the loss function *E* is designed in Equation (3).

(3) $$E(a_{k},b_{k})=\sum_{i\in w_{k}}\left({\left(a_{k}I_{i}+b_{k}-p_{i}\right)}^{2}+\varepsilon a_{k}^{2}\right)$$

where *ε* is a regularization parameter to avoid overlarge *a_k_*. The partial derivative of loss function is used to solve the optimal parameters, and expressions of *a_k_* and *b_k_* can be obtained, as shown in Formula (4). Let $\bar{p_{k}}$ and $\bar{I_{k}}$ be the average within their respective filter window and *σ_k_* be the covariance. An edge-holding approach is taken to highlight the edge features of the DR image and to weaken shadow interference, meaning that the solution to the equation is obtained by using *p* as a guide map for *I*.

(4) $$\begin{array}{l} a_{k}=\frac{\bar{p_{k}I_{k}}-p_{k}I_{k}}{\sigma_{k}^{2}+\varepsilon }\\ b_{k}=\bar{p_{k}}-a_{k}\bar{I_{k}} \end{array}\}\overset{p=I}{\to }\left\{ \begin{array}{l} a_{k}=\frac{\sigma_{k}^{2}}{\sigma_{k}^{2}+\varepsilon }\\ b_{k}=(1-a_{k}){\bar{p}}_{k} \end{array}\right.$$

Taking the mean smoothing window radius *r* = 16 and the regularization parameter *ε* = 0.01, the DR images of injection-molded parts acquired under X-ray were fast guided filtered. A DR image after fast guidance filtering is shown in Figure 2b. It can be seen that the edge details are more prominent, which demonstrates the filter’s capability of resisting the shadow interference of the workpiece under X-rays to a certain extent and reducing noise.

### 2.2. Defect and Task-Setting

**Figure 3:**
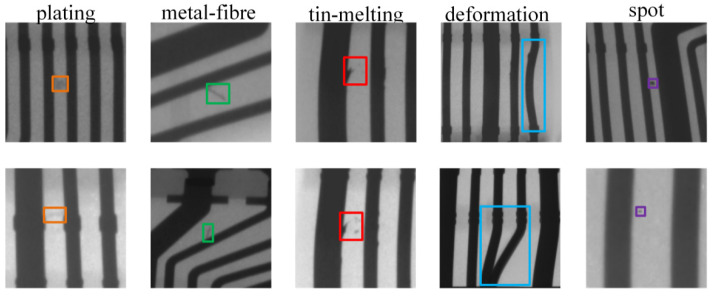
Examples of common types of defects in the insert molding under X-ray.

**Figure 4:**
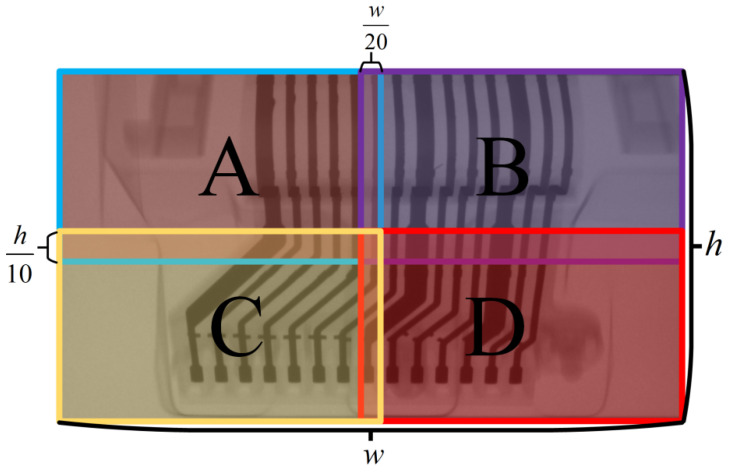
The DR images were cut by overlap. Where A, B, C and D represent the image blocks after the “image overlap” cut.

The defects are classified into five types, namely plating, metal fibers, melted tin, deformations and spots, with some typical labeled examples shown in Figure 3. It is known that the camera field of view is 1538 × 864, and the average area of each type of defect is 0.041%, 0.045%, 0.048%, 0.0094%, 1.19%, and less than 2% of the whole picture, respectively. Considering the small scale of most defects, we adopt the overlap to slice the collected images, as shown in Figure 4. Since the deformation defects are non-minor defects, the overlap width of is defined as the maximum of the other four classes of defects in the actual operation. The constructed dataset includes 2177 local cutting maps mainly used to detect plating, metal fibers, melted tin, deformations and spots, and 1522 global maps mainly used to detect all defects, all of which contain defects.

## 3. Proposed Methods

The recall and inference speed of the target are equally important in the defect detection for insert molding. With the development of target detection, the one-stage detection relies on the advantage of rapidity and is also able to match or even surpass the two-stage method in terms of accuracy, becoming the mainstream defect detection framework. Such supervised-based target detection networks can be classified as backbone, neck and head along with their functional structure. The backbone is mainly used for feature extraction and feature map generation, followed by the neck, which is charged with fusing and greatly utilizing the features extracted from the backbone to output to the head for detection tasks. Our work uses the one-stage detection network yolov5 as the basic framework. As the SOTA method of the yolo family, it performs the detection of targets in a supervised manner by regression without explicitly solving for the regional proposal. Compared with the two-stage proposal-based network, it can predict all bounding boxes and class confidence by inputting one image into the neural network at a time. With this section, we will introduce the yolo family of detection algorithms and then put forward our method.

### 3.1. The Yolo Series and Yolov5

The community divides the yolo into five versions by default, the first three of which are from its inventor. Although later versions are anchor-based object detection, yolov1 was the first to adopt anchor-free, which divides the input image into S × S grids, and whether each grid is responsible for predicting the object must be determined by its ground truth center. Given the prediction task of B bounding boxes in each grid, besides the coordinates and confidence (x, y, w, h, confidence), there is also the category information represented by the one-hot code (the total number of class is C), and the output is expressed as S × S × (5 × B + C). Following the basic framework of yolov1 with the joint training based on detection and classification, yolov2 adopts the anchor-based strategy and uses the K-means for clustering analysis of ground truth to make the model easier to learn. It designs a new feature extractor named DarkNet-19 for the backbone, reusing the ‘Network in Network’ [26] idea to reduce the model parameters by about 33% relatively at the same level of accuracy. Taking on the small target puzzle, the passthrough layer is proposed in yolov2, which utilizes high-resolution fine-grained features to connect with the low ones. Yolov3 is designed with DarkNet-53 as the backbone, which can obtain comparable accuracy with fewer parameter compared with ResNet [27]. The multi-feature layer prediction strategy of feature pyramid networks (FPN) [28] is also borrowed to enrich the grid density and enhance the multi-scale detection performance, especially adding the detection of small objects. Alexey et al. proposed yolov4, which was founded upon the yolov3 architecture and was accepted by its inventors. The network adopted the excellent optimization strategy in CNN recently, which is reflected in various aspects such as data processing (mosaic, mixup, etc.), backbone network (augmented receptive field and improved PANet, CSPNet), training techniques (self-adversarial), activation function (swish and mish), and loss function (ciou), which can be a kaleidoscope of target detection tricks. The network also shows state of the art performance on the COCO datasets, balancing accuracy and speed.

**Figure 5:**
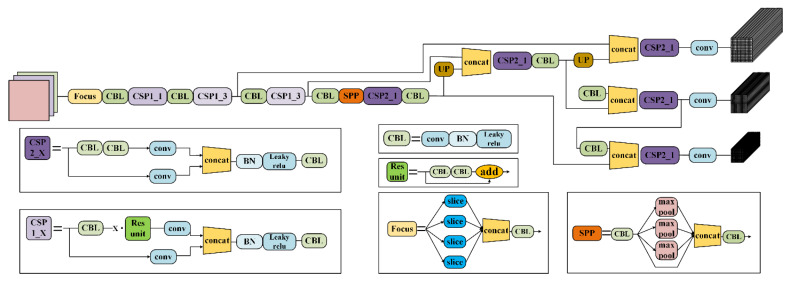
The overall structure of yolov5s.

Ultralytics released yolov5 within the following two months; however, it was mainly an improvement on yolov3, integrating a large number of computer vision technologies for easy engineering implementation. There are four releases of yolov5 according to network complexity, from simple to complex being yolov5s, yolov5m, yolov5l and yolov5x. It uses various data enhancement methods such as mosaic, adaptive anchor calculation and adaptive image scaling on the input to enrich the feature extraction of the backbone. Using *focus* in the backbone of CSPNet [26], the first feature layer is pixel-unshuffled to effectively reduce computation. At the same time, CSPNet is able to solve the problem of a repeating gradient for network optimization in the backbone of a large neural network by integrating gradient changes in the feature map, and reducing the number of model parameters and floating-point operations (FLOPs). The neck of the network adopts SPP and PANet, while the cross stage partial network is optimized to enhance the feature fusion capability, and finally three feature layers are generated on the detection head, as shown in Figure 5, which presents the overall framework of yolov5s.

### 3.2. Our Work

**Figure 6:**
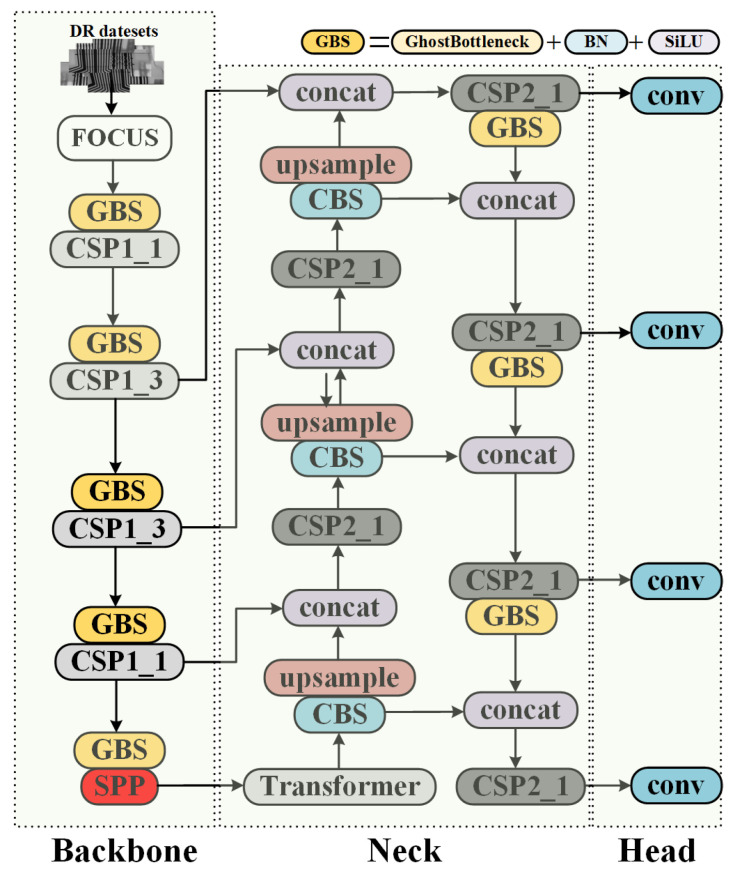
The overall structure of our proposed network.

Considering the accessibility of engineering implementation and the rapidity of detection, we performed defect detection for DR images of injection-molded workpieces with a derived lightweight deep network based on yolov5s. To further reduce the parameter of the backbone, the proposed method replaces conventional convolution with Ghost bottleneck as the main feature extraction module, and adds the transformer structure as the end feature inheritance of the backbone. The network remains to use PANet and SPP as the neck, increasing the receptive field and fusing the low-level detail features of the backbone to improve the accuracy of the bounding box. Besides using overlap for dataset construction, which increases the scale variation of training images, we added a new detection head of different sizes to further enrich the detection scale. The overall structure of our proposed network is shown in Figure 6.

#### 3.2.1. Focus Module

While extracting the image features, the reduced scale information is often concentrated in the channel space. The most common way is convolution firstly, which adjusts the size by means of suitable convolution parameter settings such as stride, padding, and the number of convolution kernels to adjust the channel depth. There is no doubt that the convolution with nonlinear feature extraction comes with training parameters, followed by pooling operations, the common types of which include polar pooling, random pooling, average pooling, etc. This bionic vision is frequently used to reduce the feature dimensionality of its output after convolutional layers. An excellent network should minimize the number of parameters to shrink the feature map while alleviating information loss in the shallow transform. Referring to the *reorg* used in yolov2 after the fine-grained feature layer, this layer splits the intermediate layer features after reducing the channels and connects them to the final feature layer with low resolution for detection, which provides a 1% improvement in the mean average precision. Feature cross-connectivity is positive, but the usefulness of directly stacking feature maps of different resolutions is ambiguous, with little gain for non-small objects. Our proposed method already performed image overlap before the backbone, so the tiny target is not a bottleneck in difficulty. We follow the *focus* module proposed by Jocher et al. to perform slicing on the input images. This module is similar to the neighborhood down sampling operation, which samples the input at every pixel interval. The four obtained images are concatenated in the third channel and then convolved to obtain a two-fold down sampled feature map with no information loss.

**Figure 7:**
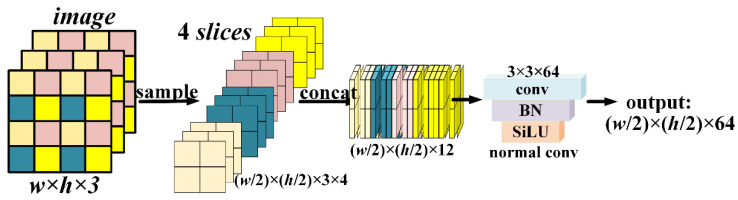
*Focus* module with parameter details.

As shown in Figure 7, the slicing deepens the number of channels and reduces the FLOPs compared to the 2× down-sampling convolution. The FLOPs’ [26] complexity is calculated as shown in Equation (5).

(5) $$\mathrm{FLOPs}=(2\times C_{i}\times K^{2}-1)\times H\times W\times C_{o}$$

where *C_i_* denotes the input channel, *K* is the size of convolution kernel, *H* and *W* are the output feature map sizes, and *C_o_* is the output channel. Taking an input of 3 × 640 × 640 as an example, and assuming that the convolution kernel size is 3 × 3, at the same time, keeping the output channel and slice output consistent, with both being 12, the number of additional parameters for the convolution operation is about 0.65 GFLOPs, while *focus* can avoid these calculations. The convolution at the tail of the *focus* is used to eliminate the stacking effect and is consistent with the convolution after 2× down-sampling. From the perspective of image information, *focus* retains the low-level image detail information better than the down-sampling of convolutional or pooling, and achieves lossless down-sampling.

#### 3.2.2. Backbone

The proposed method uses the Ghost module instead of common convolution in the backbone to perform 2× down-sampling to resize the feature maps. The main feature extractor in the backbone network is the cross-stage partial network structure proposed in yolov4, which borrows the design idea of CSPNet. It has been experimentally shown that the module can reduce the number of parameters to improve the learning ability of the convolutional neural network and reduce the computational bottleneck and memory cost. The complexity of the CSP is controlled by using *depth* and *width* in line with yolov5, so that model parameters can be flexibly adjusted. The specific composition of the backbone network is presented in Figure 6, where the training datasets, after being sliced by the *focus* module, go through four stages, all of which contain Ghost blocks and CSP blocks. The process is summarized as follows: after each stage performs 2× down-sampling of the input from the previous stage, the CSP module performs feature extraction to obtain a total of four feature maps, and in order to enrich the feature scales, a Ghost module is also used at the end of the backbone features for 2× down-sampling to obtain a total of five feature maps with different scales.

**Figure 8:**
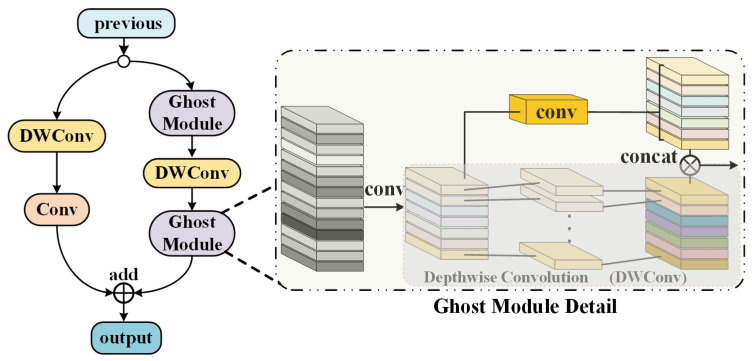
The structure of the Ghost-bottleneck module.

**Figure 9:**
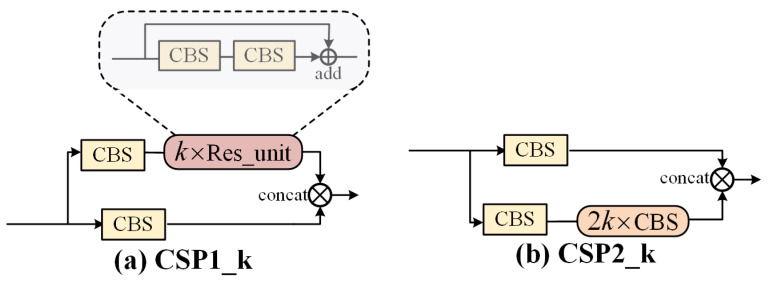
Two types of CSP blocks applied in the backbone and neck. (**a**) The CSP module for feature extraction in backbone networks (**b**) The CSP module for feature enhancement applied in the Neck of Network.

The details of Ghost-bottleneck, which replaces the regular down-sampling convolution operation, are shown in Figure 8. The feature map obtained from the previous layer goes to two branches: the left branch goes through depthwise convolution to reduce the feature scale, and then through regular convolution to double the channel; the right branch goes through the Ghost module for the convolution operation, and then down-sampling is performed by means of depthwise convolution and goes through the Ghost module again to double the channel. Finally, the two branches add up together as the output. The simplicity of this module lies in the fact that half of the feature maps are obtained by the Ghost feature linear transformation (i.e., group convolution), which results in a parameter reduction of at least 2 times compared to conventional convolution. The CSP structure is mainly applied in backbone and neck. Figure 9a shows that the CSP1_*k* implemented in the backbone contains residual blocks. Figure 9b shows CSP2_*k* implemented in the neck, where *k* denotes the number of embedded residual units or convolution blocks. This cross-stage structure splits the gradient flow into different network paths and integrates all the changes into the feature map, and thus ensuring the fusion and extraction of features while screening out duplicate gradient information to achieve the optimization of accuracy and parameter.

#### 3.2.3. Neck and Detection Head

**Figure 10:**
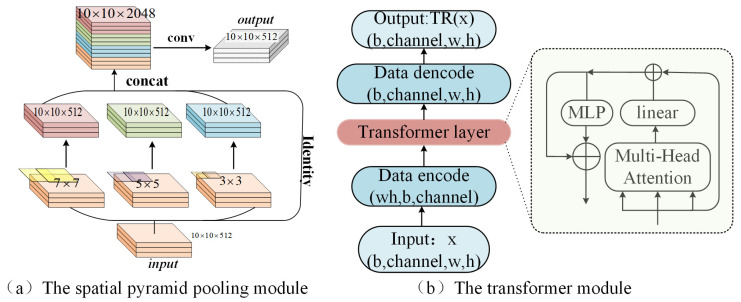
The structure of SPP module (**a**) and transformer (**b**).

To better utilize the multi-scale features extracted from backbone, we applied the SPP module and PANet structure in the neck. As shown in Figure 10a, the SPP is structured to enhance the receptive field of the network through three same pooling of 3 × 3, 5 × 5 and 7 × 7, with identity making jump connections for multiple feature fusion, which enriches the local–global association and feature map representation. In defective target detection, coordinate regression relies more on the low-level details while feature classification requires deep semantic information. This disharmony is first moderated by the FPN, and the detection effect is further enhanced by the PANet, which enhances information propagation and multiplexing of shallow features by creating a third branch that effectively fuses high-level receptive field with low-level positional information, avoiding similarity perturbations of disjoint targets in DR images, as shown in the structural detail in Figure 6. Following the four detection heads immediately below, we added higher level detection heads to form four dense detection grids, which are not a significant increase in computation, but make the network more friendly for dense small objects.

We place the transformer at the end of the backbone network, in line with the general self-attentive module. This module is located after the SPP module, and firstly, the input features are encoded in channels, which are transformed 4 times linearly to obtain 4-head, respectively. After the scaled dot-product attention is performed separately for each group, integrated by multi-layer perceptron, the data are decoded into the same format as the input.

#### 3.2.4. Loss Function

**Figure 11:**
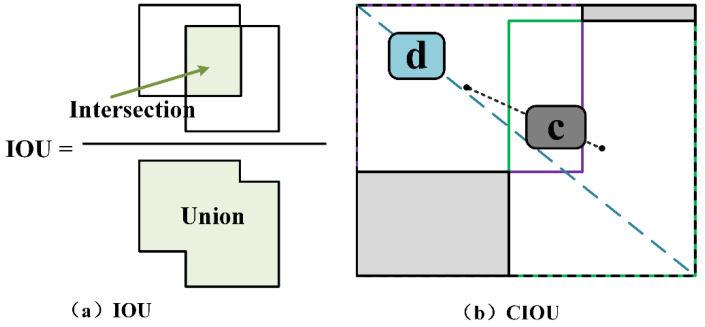
The principle of IOU (**a**) and CIOU (**b**).

The loss function of the proposed method consists of three main components: classification, confidence and regression. Losses in both classification and confidence are calculated by the BCEWithLogitsLoss, which combines the binary cross-entropy loss with sigmoid for the operation. The regression is measured by the ciou (complete-iou) loss, which comes from calculating the ciou between the predicted and real boxes. Compared to the intersection over union (iou), it is more likely that ciou will take into account the similarity of scale information such as overlap, central distance and aspect ratio between the predicted and real borders. The schematic diagram of ciou is shown in Figure 11.

With a penalty added to iou, the ciou minimizes the normalized distance of the center between the prediction *b* and *gt b^gt^*, resulting in a significant reduction in convergence. The formula is shown in (6), where alpha is the weight function, and *v* is defined as a function of evaluating the consistency of the aspect ratio, calculated by the discrepancy in width and height between *b* and *b^gt^*. d is the Euclidean distance of center between *b* and *b^gt^*. *c* is the diagonal distance of closure. The iou is the iou calculation function.

(6) $$\begin{array}{l} R_{ciou}=\frac{d^{2}(b,b^{gt})}{c^{2}}+\alpha \cdot v\\ \alpha =\frac{v}{(1-iou(b,b^{gt}))+v}\\ \;v=\frac{4}{\pi^{2}}\times {\left(\arctan\frac{w^{gt}}{h^{gt}}-\arctan\frac{w}{h}\right)}^{2} \end{array}$$

**Figure 12:**
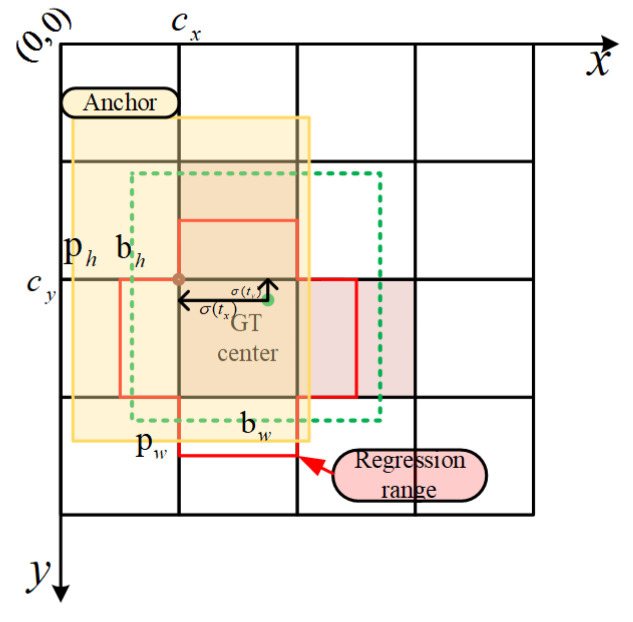
Definition of positive samples in the training process.

The calculation rule for the positive sample anchor is updated to cross-grid matching as follows: firstly, for any output layer, the max-iou matching is replaced by the shape rule, which calculates the aspect ratio of the bounding box (bbox) to the current layer anchor, and sets a threshold t to screen out prediction boxes with large deviations to mark them as background. Secondly, index the grid that the bbox obtained in the previous belongs to, with the two grids closest to the center of ground truth (*gt*) selected so that these three grids are used to predict the bbox. This process is shown in Figure 12, where the green point in a single detection feature layer is the *gt* center and the red boundary is the regression range of the coordinate. The top and right grids were chosen to be responsible for predicting this *gt* as well, depending on the distance from the adjacent grid to the green point, and together with the grid where the center of the *gt* is located, the number of positive samples is three times as large as before. Different from the previous version of yolo, the *gt* could obtain more positive samples on multiple prediction layers, and the matching base of each increased by two times. Combined with the shape rule, this method multiplies the number of positive samples, which further promoted the network’s convergence.

The bounding box regression (*b_x_*, *b_y_*, *b_w_*, *b_h_*) is defined by Formula (7), where (*c_x_*, *c_y_*) is the upper left vertex of the gt grid, (*t_x_*, *t_y_*) is the gt center offset, *t_w_* and *t_h_* are the *gt* width and height offsets, respectively, and *p_w_* and *p_h_* denote the width and height of the anchor. The range of the bounding box regression center is (−0.5~1.5), which corresponds to the red bounding box in Figure 12.

(7) $$\begin{array}{l} b_{x}=2\sigma (t_{x})-0.5+c_{x}\\ b_{y}=2\sigma (t_{y})-0.5+c_{y}\\ b_{w}=p_{w}{\left(2\sigma (t_{w})\right)}^{2}\\ b_{h}=p_{h}{\left(2\sigma (t_{h})\right)}^{2} \end{array}$$

*S* × *S* denotes the grid of the detection layer, and *B* denotes the number of bounding boxes in each grid. The boxes containing the ground truth center of the object are counted before training, and classification and confidence loss are calculated for these boxes. The rest of the boxes that do not contain objects only calculate the confidence loss. Both classification loss and confidence loss are calculated using binary cross-entropy, which is calculated in Formula (8).

(8) $$\begin{array}{l} L_{all}=L_{ciou}+L_{conf}+L_{cls}\\ L_{ciou}=1-iou(b,b^{gt})+R_{ciou}\\ L_{conf}=-\sum_{i=0}^{S\times S}\sum_{j=0}^{B}I_{ij}^{obj}[C_{i}^{*}\log(C_{i})+(1-C_{i}^{*})\log(1-C_{i})]\\ \;-\lambda_{noobj}\times \sum_{i=0}^{S\times S}\sum_{j=0}^{B}I_{ij}^{noobj}[C_{i}^{*}\log(C_{i})+(1-C_{i}^{*})\log(1-C_{i})]\\ L_{cls}=-\sum_{i=0}^{S\times S}I_{ij}^{obj}\sum_{c\in classes}\left[p_{i}^{*}(c)\log(p_{i}(c))+(1-p_{i}^{*}(c))\log(1-p_{i}(c))\right] \end{array}$$

where *λ_noobj_* denotes the weight coefficient calculated for the loss without the object, which is used to reduce its contribution. *I_ij_* denotes whether the object exists in the *j*-th bounding box of the *i*-th grid. C* denotes the confidence level: 1 if the box of the gride cell contains the object responsible for the prediction, and 0 otherwise. *C* denotes the prediction confidence level. P* is the true probability value (1 or 0) and *P* is the predicted classification probability.

## 4. Experiment and Analysis

After the DR image pre-processing including fast guided filtering and image overlap, the proposed defect detection network in the previous section is then implemented in PyTorch. Peer algorithms such as classical two-stage algorithms (Faster RCNN, etc.) and traditional yolo series (yolov4, etc.) are carried out on the same X-ray image datasets for comparison. In addition, the modular ablation experiments are also conducted to verify the superior performance of the introduced new structures in the proposed network.

### 4.1. Datasets

A total of 3699 DR images containing defective targets were obtained after pre-processing, including the overlap cut and the origin image, which were 784 × 476 and 1636 × 864 in size, respectively. The data were randomly divided between the training set and the test set at a ratio of 3:1 approximately, and the results of the division are shown in Table 1.

### 4.2. Evaluating Indicator

The results can be categorized according to the model’s predictions on the test set compared to the labeled GT: both actual and predicted positive (true positive, *TP*), actual negative but predicted positive (false positive, *FP*), actual positive but predicted negative (true negative, *TN*), and both actual and predicted negative (false negative, *FN*). The detection algorithms are often evaluated in terms of accuracy and speed. The accuracy is measured by precision, recall average and *AP*, and speed is measured by model size and the inference time for each image. Precision indicates the proportion of *TP* to all predicted positives, and recall indicates the proportion of *TP* to all positive targets. For each category, the *AP* value (average precision) is the integral of the *P*–*R* curve between (0, 1), and the average of the *AP* for all categories is the mean average precision (*mAP*), which is calculated as in Equation (9).

(9) $$\begin{array}{l} P=\frac{TP}{TP+FP}\;R=\frac{TP}{TP+FN}\\ AP=\int_{0}^{1}p(r)dr\;mAP=\frac{1}{n}\sum_{i=1}^{n}AP_{i} \end{array}$$

### 4.3. Experimental Details

The implementing platform is based on a computer with an Intel i7-9700K CPU, 64 G RAM, 2 × 11 G Titan V GPU and a 2 T SSD hard drive inside. The computer is installed with an Ubuntu 18.04 operating system and also the CUDA-10.1 and cudnn-7.6.2. PyTorch 1.8 is chosen as the deep learning framework and Python 3.7 is used as the programming language. In the peer network comparison, we selected some classical excellent target detection algorithms, including both one- and two-stage methods. The two-stage methods include faster RCNN, R-FCN, FPN, NAS-FPN, and cascade RCNN, while the one-stage methods include yolov3~yolov5. The ablation experiments for module evaluation are carried out based on yolov5. The basic parameter settings of the implementation are as follows: the pre-trained network weights using ImageNet, a batch size of four, the SGD optimization algorithm with momentum of 0.937, an initial learning rate of 0.01, iterations of 300 epochs, a positive sample threshold of four sets in yolo5, and gains of 0.05, 0.5 and 1.0 for localization, classification and confidence, respectively. All of the network trainings use the pre-processed image dataset.

### 4.4. Experimental Results

#### 4.4.1. Peer Comparison

**Figure 13:**
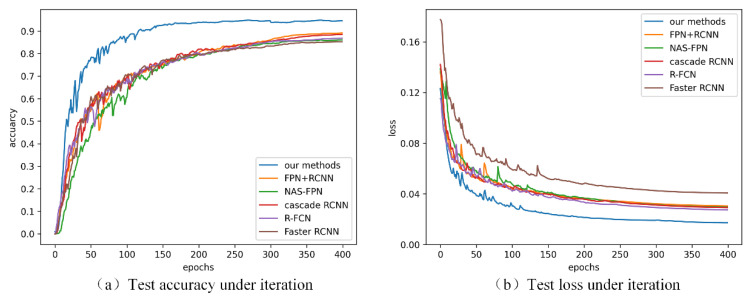
Test accuracy (**a**) and loss (**b**) of the proposed method and some typical two-stage detection algorithms in the DR datasets.

**Figure 14:**
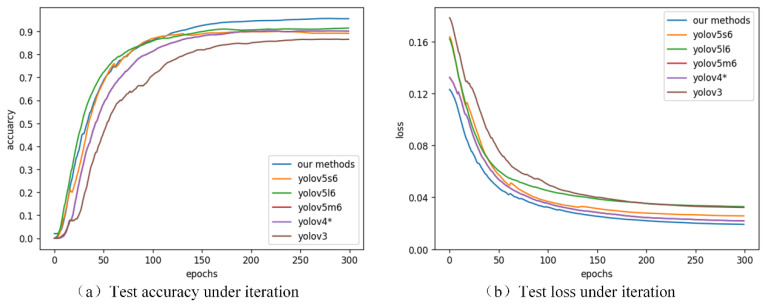
Test accuracy (**a**) and loss (**b**) curves for the proposed method and the yolo family of detection algorithms in the DR datasets.

The accuracy and loss of the proposed method tested with the two-stage network during training are shown in Figure 13, and the comparison with the one-stage method is presented in Figure 14. It can be seen from the figure that each network converged and the accuracy of the test remained stable. The one-stage methods generally converge faster, especially the yolov5 series. Details of precision, recall, *mAP* and inference speed are listed in Table 2 and Table 3.

**Figure 15:**
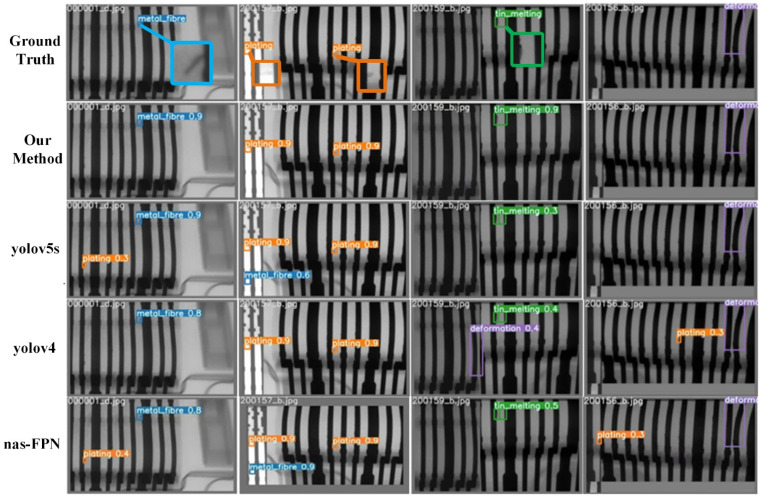
Comparison of the actual detection results of the experimental networks.

According to the evaluation presented in Table 2 and Table 3, it can be seen that one-stage detection methods tend to be lighter and faster than two-stage methods, with high accuracy rates, despite the slightly higher recall of some two-stage networks. Our improved method based on yolov5 corrects this drawback, and the accuracy and recall are significantly higher, reaching the optimal *mAP* on the DR datasets. In the s-version of the network, the proposed method increases 5% in *mAP* over the yolov5s6, and the model size is reduced by 32% with the same four detection heads. The inference time on CPU is also recorded in Table 3, which is nearly 20 times slower than on GPU. We selected NAS-FPN (the best performer in the second stage), yolov4, yolov5s6 and the proposed method to compare the actual detection effect, and the results are displayed in Figure 15. It can be seen that the proposed method possesses a better performance in terms of the confidence and regression box. Even in the mosaic’s local splice map, it carries few false detections.

#### 4.4.2. Ablation Experiments

With outstanding advantages in accuracy and speed compared to peer networks, the proposed method validates the superior performance of our study for defect detection on the DR datasets. To further validate the effectiveness of the network composition and key modules, we performed ablation experiments on the proposed method based on the DR datasets with items including the *focus* module, transformer, Ghost, etc., together with the idea of control variables. The ablation experiments are built on the consistent operating environment mentioned above, and the detailed evaluation results are shown in Table 4.

From the results of the ablation experiments, we can see that the increase in the number of detection layers and the transformer have a high enhancement effect on the network’s performance in terms of *mAP*, and the Ghost module can effectively reduce the number of parameters. It is worth mentioning that the transformer increases performance at the cost of low inference time. Using the multiscale training method did not significantly improve network performance, and we speculate that this is duplicated by the effect of overlapping. Additionally, focus did not reduce the model size, but was able to effectively reduce FLOPs. Without fancy functional-type module embedding, the network still owns strong feature representation and faster inference speed, while it cannot meet the practical requirements, which is confirmed by the experimental results in ablation (7). The pre-processing is also proven to contribute, without which there is a 7% drop in *mAP*.

## 5. Discussion

Our study explores the application of target detection for manufacturing defect detection on the DR images of automotive electronic injection-molded workpieces, and proposes detection algorithms that are easy to implement while obtaining promising results. The deployment of detection relies on the lightness and convenience of the model, which has been done quite well in the original yolov5. The proposed method makes further enhancements, reducing the model parameters by 32%, mainly due to our introducing of the Ghost module.

### 5.1. Why Choose Target Detection to Do Defect Detection?

It is not intentional to ignore other deep learning methods, nor is it a random se-lection of target detection algorithms as the basic framework for DR image defect detection experiments. We mainly consider the irregular shape of the majority of defect targets in this study, such as the non-uniform distribution of molten tin and the narrow length of metal wires, and it is ambiguous and difficult to label them pixel by pixel, which makes the supervised segmentation-based algorithms difficult to perform. Some unsupervised methods [29,30] tend to be more suitable for plain backgrounds and non-small target defects, while injection-molded artifacts can form shadow interference of indeterminate direction under X-rays, which is not conducive to detection implementation.

### 5.2. Network Complexity and Performance

**Figure 16:**
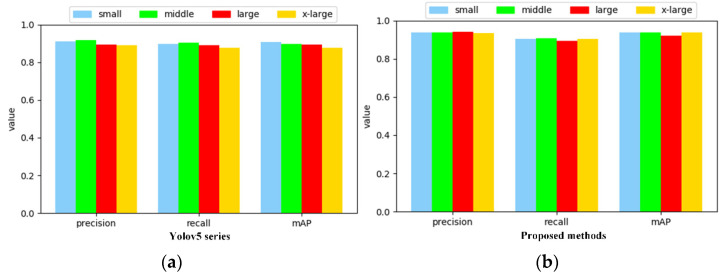
Comparison of DR image detection performance of yolov5 (**a**) with the proposed method (**b**) in deeper and wider networks.

It is already known that yolov5 has the flexibility to adjust the depth and width of the network through the parameters of *depth* and *width*. Whereas *depth* controls the network depth, which is implemented by the complexity of the CSP-Bottleneck, and it is further determined by the number of residual and convolutional blocks in the CSP, *width* controls the network width, which is implemented by adjusting the number of convolutions. Assuming that the *depth* and *width* are both 1, the basic number of the residual and convolutional blocks in CSP is 6, and the basic number of convolutional kernels is 64. The proposed method uses the most compact structure with depth of 0.33 and width of 0.5, and the corresponding number of bases are 0.33 × 6 and 0.5 × 64, respectively. We explored the performance of wider and deeper networks for DR image defect detection based on the proposed method. It is shown in Figure 16 that the proposed method is compared with the yolov5 series in networks of different depths and widths, where *small* denotes the baseline network structure, *middle* has a depth and width of (0.66, 0.75), *large* has a depth and width of (1, 1), and *x-large* has a depth and width of (1.33, 1.25), respectively.

It can be seen from Figure 16 that the proposed method is more stable in terms of performance, and mAP does not decay significantly as the network deepens and widens. However, it does not increase obviously, indicating that the shallower semantics is sufficient for the target feature representation, and the increased complexity brings computational gains that often outweigh the losses in speed and lightweight.

### 5.3. Robustness Test and Analysis

**Figure 17:**
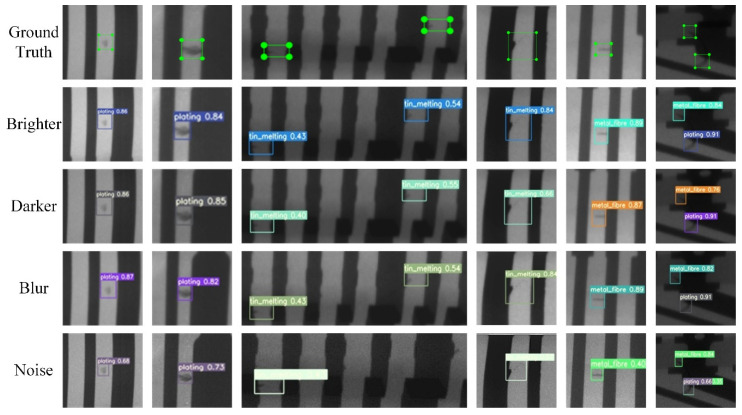
The DR image defect detection under different interference. Brighter (l = 0.2), darkened (l = 0.8), blur (η = 0.2), salt and pepper noise (σ = 0.02).

It is not sensitive to detect unstable imaging, which is a common problem of supervised defect detection. Although our algorithm achieves qualified detection performance in the test set, verification of its robustness and generalization is also necessary. The detection effects of the test set after luminance change, image blurring, and the addition of pretzel noise [31,32,33] are shown in Figure 17. Their *mAP* average fluctuation is statistically less than 3%, so the overall robustness test is of a qualified level. Observing the visual results, it is found that noise interference and blurring have a greater impact on the imaging quality and lower confidence of the regression frame, which indicates that both are more likely to destroy the image features and need to be highlighted in the production practice.

**Figure 18:**
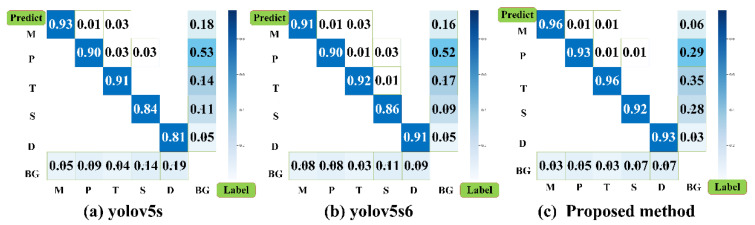
Confusion matrix results for detection. (**a**) Confusion matrix of detection results for yolov5s. (**b**) Confusion matrix of detection results for yolov5s6. (**c**) Confusion matrix of detection results for our proposed method.

According to the evaluation metrics, the proposed method excels in lightweight and inference speed, and its robustness is also appreciable, meeting the needs of industrial applications. It still needs to be further explored for its applicability, although our method achieves optimal results for peer networks on *mAP*. We visualized the confusion matrix results for analyzing detection misses regarding yolov5s, yolov5s6 and the proposed method, as shown in Figure 18.

**Figure 19:**
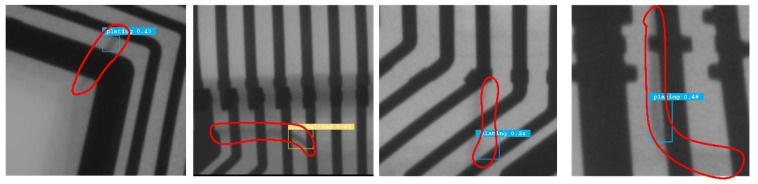
Some examples of typical misjudgments.

It can be seen that the majority of detection errors tend to focus on false positives, where background interference is misclassified as a defect, along with samples that are detected but misclassified, while the proportion of true negatives is small in comparison. Combined with some inspection examples, as shown in Figure 19, it is difficult to be sure of the authenticity of the defect manually in the local field of view, while extending the field of view will further determine whether it is a defect or interference. Therefore, we reasoned that having prior knowledge and a larger receptive field will help to exclude such interference, which will be explored in further study.

## 6. Conclusions

In this study, we designed a DR image defect detection algorithm based on yolov5 for detecting insert molding in automotive electronics. Firstly, the window level and window width are adjusted in the pre-processing stage of the acquired data and edge retention is performed using fast guided filtering. The overlap is taken to handle the detection of tiny target anomalies and construct a multi-task dataset. Secondly, the backbone network with feature enhancement is improved to further achieve the reduction in the number of parameters by Ghost, replacing the standard convolutional network. Meanwhile, CSP-module is embedded in the backbone and neck to enhance the feature extraction of the network. The transformer attention module is added after the spatial pyramid pooling to map the data to the subspace to avoid over-fitting while reducing the computational effort. Finally, the consistent experiments are performed with typical two-stage networks and original yolo series target detection algorithms based on the DR datasets.

The experimental results show that our proposed method has the best performance in terms of accuracy, recall, and *mAP* for DR images of the automotive electronic insert molding. The test speed is 0.014 s, which meets the applicable requirements and is higher than other peer networks, and the number of model parameters is reduced by 32% compared to yolov5s6. Ablation experiments are conducted on the internal structure of the network to deconstruct the gain of the key modules of the algorithm, verifying the effectiveness of the transformer and multi-level detection in improving accuracy, and that Ghost is beneficial to reduce the number of parameters. Finally, we conducted robustness analysis and the reliable verification to verify the anti-interference capability of the model. The results indicate that the network has an excellent detection performance even under unstable imaging, such as brightness fluctuations, blurring and noise interference. The missed detection and application possibilities of defective recalls are discussed, showing that the proposed method can meet the application requirements. The series of experimental results show that our proposed method can effectively detect anomalies in DR images, and also provide a reference for other internal imaging visual inspection.

## Figures and Tables

**Table 1:** Details of data division in DR image of injection parts.

Size	Train Set	Test Set
784 × 476	1707	470
1636 × 864	1163	359
Total	2870	829

**Table 2:** Performance of peer defect target detection algorithms on the RR dataset (mainly in the two-stage).

Network	P	R	*mAP*	Inference/s	Size/MB
Faster RCNN	0.783	0.864	0.832	0.095	378.5
R-FCN	0.790	0.865	0.835	0.0625	402
FPN	0.818	0.913	0.897	0.086	501
NAS-FPN	0.831	0.927	0.906	0.173	726
Cascade RCNN	0.822	0.884	0.894	0.087	425
Our methods	0.938	0.903	0.936	0.014	17.1

**Table 3:** Performance of peer defect target detection algorithms on the RR dataset (the one-stage).

Network	P	R	*mAP*	Inference/ms (GPU/CPU)	Size/MB
yolov3-spp	0.883	0.896	0.897	16.8 (341)	125.7
yolov4	0.9118	0.8966	0.9027	36.2 (569)	256.4
Yolov5s	0.879	0.833	0.835	8.4 (175)	14.4
yolov5s6(4-layer)	0.9109	0.868	0.868	11.2 (264)	25.2
Our methods	0.938	0.903	0.936	14.0 (283)	17.1

**Table 4:** Ablation experiments of the proposed method.

Module	(1)	(2)	(3)	(4)	(5)	(6)	(7)	(8)
Pre-process	y	Y	Y	y	y	y	y	
4-layer	y	Y	y	y	y	y		
SPP	y	Y	y	y	y			
Focus	y	Y	y	y				
Multi-scale	y	Y	y					
Transformer	y	Y						
Ghost	y							
*mAP*	0.936	0.931	0.915	0.912	0.910	0.884	0.855	0.793
Size(/MB)	17.1	25.2	25.2	25.2	25.2	23.9	14.1	14.1
Speed(/ms)	11.4	10.3	10.1	10.1	10.6	10.4	8.5	8.5

## Data Availability

The raw data required to reproduce these findings cannot be shared at this time as the data also form part of an ongoing study. Once the article has been accepted, we will be glad to disclose the datasets and source code.

## References

[1] Huang G., Li X., Wu X., Li J. Application of core-pulling mechanism in injection mould design. Proceedings of the 2009 International Conference on Industrial Mechatronics and Automation (ICIMA 2009).

[2] Duan Y., Coatrieux G., Shu H., Mines-telecom I., Bretagne T., Latim I.U. Identification of digital radiography image source based on digital radiography pattern noise recognition. Proceedings of the 2014 IEEE International Conference on Image Processing (ICIP 2014).

[3] Yu H., Wei J., Zhao X., Ma Y., Chen L. The perspective detection of the X-ray digital radiography for the electrical equipment. Proceedings of the 2012 International Conference on High Voltage Engineering and Application (ICHVE 2012).

[4] Baygin M., Karakose M., Sarimaden A., Akin E. Machine vision based defect detection approach using image processing. Proceedings of the 2017 International Artificial Intelligence and Data Processing Symposium (IDAP 2017).

[5] Li F., Hang Z., Yu G., Wei G., Xinyu C. The method for glass bottle defects detecting based on machine vision. Proceedings of the 2019 Chinese Control And Decision Conference (CCDC 2019).

[6] Zhao W., Huang H., Li D., Chen F., Cheng W. (2020). Pointer defect detection based on transfer learning and improved CASCADE-RCNN. Sensors.

[7] Wu Z., Chen Y., Zhao B., Kang X., Ding Y. (2021). Review of weed detection methods based on computer vision. Sensors.

[8] Hu S., Jiabin Z., Bohao Z., Wei Z. (2021). Review of research on the inspection of surface defect based on visual perception. Comput. Integr. Manuf. Syst..

[9] Zhao Y., Shen K. (2016). Improved LBP used for detection of defects of DR images. Comput. Appl. Eng. Educ..

[10] Xiao H., Ao B. (2017). Research on Automatic Detection and Recognition System of Weld Defects in X-ray DR Image. https://kns.cnki.net/KCMS/detail/detail.aspx?dbname=CMFD201801&filename=1017711303.nh.

[11] Padilla R., Netto S.L., da Silva E.A.B. A Survey on Performance Metrics for Object-Detection Algorithms. Proceedings of the 2020 International Conference on Systems, Signals and Image Processing (IWSSIP 2020).

[12] Chen J., Li Y., Zhao J. X-ray of Tire Defects Detection via Modified Faster R-CNN. Proceedings of the 2019 2nd International Conference on Safety Produce Informatization (IICSPI 2019).

[13] Biao C. (2020). Research on defect detection of X-ray DR images of casting based on Mask R-CNN. Chin. J. Sci. Instrum..

[14] Girshick R., Donahue J., Darrell T., Malik J. Rich feature hierarchies for accurate object detection and semantic segmentation. Proceedings of the 2014 IEEE Conference on Computer Vision and Pattern Recognition (CVPR 2014).

[15] Girshick R. Fast R-CNN. Proceedings of the 2015 IEEE International Conference on Computer Vision (ICCV 2015).

[16] Ren S., He K., Girshick R., Sun J. (2017). Faster R-CNN: Towards Real-Time Object Detection with Region Proposal Networks. IEEE Trans. Pattern Anal. Mach. Intell..

[17] He K., Gkioxari G., Dollár P., Girshick R. (2020). Mask R-CNN. IEEE Trans. Pattern Anal. Mach. Intell..

[18] Redmon J., Divvala S., Girshick R., Farhadi A. You only look once: Unified, real-time object detection. Proceedings of the 2016 IEEE Conference on Computer Vision and Pattern Recognition (CVPR 2016).

[19] Redmon J., Farhadi A. YOLO9000: Better, faster, stronger. Proceedings of the 2017 IEEE Conference on Computer Vision and Pattern Recognition (CVPR 2017).

[20] Redmon J., Farhadi A. (2018). Yolov3: An incremental improvement. arXiv.

[21] Bochkovskiy A., Wang C.Y., Liao H.Y.M. (2020). YOLOv4: Optimal Speed and Accuracy of Object Detection. arXiv.

[22] Liu W., Anguelov D., Erhan D., Szegedy C., Reed S., Fu C.Y., Berg A.C., Leibe B., Matas J., Sebe N., Welling M. (2016). SSD: Single shot multibox detector. Computer Vision—ECCV 2016.

[23] Su Y., Yan P. A defect detection method of gear end-face based on modified YOLO-V3. Proceedings of the 2020 10th Institute of Electrical and Electronics Engineers International Conference on Cyber Technology in Automation, Control, and Intelligent Systems (CYBER 2020).

[24] He K., Sun J., Tang X. (2013). Guided image filtering. IEEE Trans. Pattern Anal. Mach. Intell..

[25] He K., Sun J. (2015). Fast Guided Filter. arXiv.

[26] Lin M., Chen Q., Yan S. Network in network. Proceedings of the 2nd International Conference on Learning Representations (ICLR 2014).

[27] He K., Zhang X., Ren S., Sun J. Deep residual learning for image recognition. Proceedings of the 2016 IEEE Conference on Computer Vision and Pattern Recognition (CVPR 2016).

[28] Lin T.-Y., Dollár P., Girshick R., He K., Hariharan B., Belongie S. Feature pyramid networks for object detection. Proceedings of the 2017 IEEE Conference on Computer Vision and Pattern Recognition (CVPR 2017).

[29] Bergmann P., Fauser M., Sattlegger D., Steger C. Uninformed students: Student-teacher anomaly detection with discriminative latent embeddings. Proceedings of the 2020 IEEE/CVF Conference on Computer Vision and Pattern Recognition (CVPR 2020).

[30] Dehaene D., Frigo O., Combrexelle S., Eline P. (2020). Iterative energy-based projection on a normal data manifold for anomaly localization. arXiv.

[31] Sheikh H.R., Bovik A.C. (2004). Image information and visual quality. IEEE Trans. Image Process..

[32] Fu G., Sun P., Zhu W., Yang J., Cao Y., Yang M.Y., Cao Y. (2009). A deep-learning-based approach for fast and robust steel surface defects classification. Opt. Lasers Eng..

[33] Song K., Yan Y. (2013). A noise robust method based on completed local binary patterns for hot-rolled steel strip surface defects. Appl. Surf. Sci..

